# RNA sequencing of glioblastoma tissue slice cultures reveals the effects of treatment at the transcriptional level

**DOI:** 10.1002/2211-5463.13353

**Published:** 2021-12-29

**Authors:** Susann Haehnel, Michael Rade, Nicole Kaiser, Kristin Reiche, Andreas Horn, Dennis Loeffler, Conny Blumert, Felicitas Rapp, Friedemann Horn, Juergen Meixensberger, Christof Renner, Wolf Mueller, Frank Gaunitz, Ingo Bechmann, Karsten Winter

**Affiliations:** ^1^ Institute of Anatomy Faculty of Medicine University of Leipzig Germany; ^2^ Department of Diagnostics Fraunhofer Institute of Cell Therapy and Immunology Leipzig Germany; ^3^ GSI Helmholtzzentrum für Schwerionenforschung GmbH Darmstadt Germany; ^4^ Institute of Clinical Immunology Faculty of Medicine University of Leipzig Germany; ^5^ Department of Neurosurgery University Hospital Leipzig Germany; ^6^ Department of Neurosurgery City Hospital Dessau Germany; ^7^ Department of Neuropathology University Hospital Leipzig Germany

**Keywords:** glioblastoma multiforme, radiochemotherapy, RNA sequencing, tissue slice cultures

## Abstract

One of the major challenges in cancer research is finding models that closely resemble tumors within patients. Human tissue slice cultures are a promising approach to provide a model of the patient's tumor biology *ex vivo*. Recently, it was shown that these slices can be successfully analyzed by whole transcriptome sequencing as well as automated histochemistry, increasing their usability as preclinical model. Glioblastoma multiforme (GBM) is a highly malignant brain tumor with poor prognosis and little is known about its genetic background and heterogeneity regarding therapy success. In this study, tissue from the tumors of 25 patients with primary GBM was processed into slice cultures and treated with standard therapy (irradiation and temozolomide). Total RNA sequencing and automated histochemistry were performed to enable analysis of treatment effects at a transcriptional and histological level. Slice cultures from long‐term survivors (overall survival [OS] > 24 months) exhibited more apoptosis than cultures from patients with shorter OS. Proliferation within these slices was slightly increased in contrast to other groups, but not significantly. Among all samples, 58 protein‐coding genes were upregulated and 32 downregulated in treated vs. untreated slice cultures. In general, an upregulation of DNA damage‐related and cell cycle checkpoint genes as well as enrichment of genotoxicity pathways and p53‐dependent signaling was found after treatment. Overall, the current study reproduces knowledge from former studies regarding the feasibility of transcriptomic analyses and automated histology in tissue slice cultures. We further demonstrate that the experimental data merge with the clinical follow‐up of the patients, which improves the applicability of our model system.

## Graphical abstract

In this study, we investigated mRNA expression and histological effects of radiochemotherapy treatment on freshly resected and cultivated tissue slices of 25 patients diagnosed with primary glioblastoma. We demonstrate that the experimental data obtained from these slice cultures can be correlated to the clinical course of a particular patient, thereby increasing their suitability as a preclinical model system.

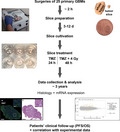

**Abbreviations**
DEG: differentially expressed gene
GBM: glioblastoma multiforme
Gy: gray
HR: hazard ratio
lfc: log2‐fold‐change
MGMT: O‐6‐Methylguanin‐DNA‐Methyltransferase
NK: natural killer cells
OS: overall survival
PFS: progression‐free survival
TMZ: temozolomide

Glioblastoma multiforme (GBM) is the most frequent malignant brain tumor in adults [1]. As astrocytoma of grade IV, it is characterized by infiltrative growth, high mitotic activity, microvascular proliferation, and necrosis [2, 3]. Despite aggressive standard combination therapy of surgical resection, irradiation, and chemotherapy with temozolomide (TMZ), the median overall survival (OS) of patients with primary GBM still is only about 15 months [4, 5]. Primary GBM is defined as *de novo* development of the tumor without any evidence of a less malignant precursor tumor, whereas secondary GBMs evolve from the progression of lower grade astrocytomas [6]. Both classes differ significantly regarding their molecular evolution and genetic pathways [7], RNA expression patterns [8, 9], and the patients' prognosis and therapy response [10]. In the present study, we focused on the analysis of primary GBM.

One of the major challenges in GBM therapy is its high intra‐ and intertumoral heterogeneity and the related difficulty of predicting a patients' response to therapy [11, 12]. Many trials are aiming at the identification of predictive biomarkers, but the clinical relevance is often limited and the development of targeted drugs is still challenging [12, 13, 14, 15], not least due to the impermeability of the blood–brain barrier [16, 17]. Even the methylation status of the O‐6‐Methylguanin‐DNA‐Methyltransferase (MGMT) promotor, which is well‐established and has been shown to be related to the therapy response and prognosis, leads to heterogeneous responses in patients [18]. In 2010, four molecularly defined subgroups of GBM were established—classical, mesenchymal, proneural, and neural subtype—including characteristic gene expression patterns [19]. Despite this increase in knowledge about genetic and transcriptomic features of GBM since the implementation of TMZ‐based radiochemotherapy, the standard of care is not considerably influenced [20]. Further, even within each subtype, there still is a high intratumoral heterogeneity on the expression level [21] which underlines the strong need to develop an individualized approach for each single GBM patient.

Generally, cancer research requires model systems as realistic and as close to the original patient as possible. In recent studies, slice cultures from tumor tissue, for example, head and neck squamous cell carcinoma [22], colorectal carcinoma [23], gastric and esophagogastric junction cancer [24], and GBM [25, 26], have been shown to be a promising alternative to conventional cell culture or animal models. Slice cultures overcome interspecies differences, which often limit the translation of animal models into a clinical setting. Further, they offer a higher complexity and are closer to the *in vivo* situation than cell culture models. For GBM, the usability of such models could be enhanced by total RNA sequencing and the quantification of treatment effects within this method has been improved by the automation of histological staining analysis [26]. In the study presented here, the model system was further investigated regarding the reproducibility of the achieved results among a larger cohort of GBM patients, thereby also addressing the intra‐ and intertumoral heterogeneity. Samples from 25 patients with primary GBM were processed into slice cultures, subjected to standard radiochemotherapy, and the total RNA was sequenced in treated versus untreated slices. Concomitantly, histological analyses were performed to correlate the results from distinct methods and to evaluate the preservation of the tissue throughout the cultivation and treatment period.

## Material and methods

### Patients and samples

Glioblastoma tissue samples were obtained by surgery of 16 male and 9 female patients diagnosed with primary glioblastoma (GBM, WHO grade IV). The patient data including PFS and OS are summarized in Table S1. Surgery and diagnosis were performed at the Department of Neurosurgery and the Department of Neuropathology, University Hospital Leipzig, Germany, and at the Department of Neurosurgery, City Hospital, Dessau, Germany, according to the EANO guideline for the diagnosis and treatment of anaplastic gliomas and glioblastoma [27]. All tissue samples were subjected to organotypic tissue slice cultures and replicate number ranged from 1 to 3 depending on the amount of tissue available for the cultivation. Tissue acquisition and experimental procedure were approved by the institutional research ethics board (Ethical Review Committee of the Medical Faculty of the University of Leipzig, #144/08‐ek; registration numbers: IORG0001320, IRB00001750) and the ethic board of the Ärztekammer Sachsen‐Anhalt, Halle (Saale) in accordance with the Helsinki Declaration (https://www.wma.net/policies‐post/wma‐declaration‐of‐helsinki‐ethical‐principles‐for‐medical‐research‐involving‐human‐subjects/). The patients provided written informed consent for experimental usage of their tissue samples and retrospective analysis of the data according to the General Data Protection Regulation of the European Community (https://gdpr‐info.eu/).

### Tissue slice preparation

Tissue slices were prepared according to a previously described protocol [25, 26]. The slices were cultivated on a liquid/air interface in a humidified incubator at 37 °C and 5% CO_2_ for 6–15 days in total and provided with fresh medium every 2–3 days.

### Treatment of tissue slices

After 3–12 days in culture, slices were treated with temozolomide (TMZ, 200 µm) and X‐irradiation (4 Gy) according to a previously described protocol [26]. In brief, 24 h after the initial treatment with TMZ, x‐irradiation was performed with a 200 kV irradiation machine (Gulmay Medical D3000, Gulmay, Surrey, UK) with a copper filter. The dose rate was 1.156 Gy·min^−1^ and each sample was irradiated 3.46 min to reach the target dose of 4 Gy. Control samples were sham‐irradiated.

### Histology

Histological staining of Ki67 and TUNEL assays were done according to a previously described protocol [26].

### Imaging and image analysis

Imaging of immunofluorescently stained microscope slides and image analysis of respective images was performed using previously described methods [26]. In brief, microscope slides were fully digitized at 20× magnification using a digital slide scanner (Pannoramic Scan II, 3D HISTECH Ltd., Budapest, Hungary) equipped with a quad band (DAPI/FITC/TRITC/Cy5) filter set and PNG images were exported from slide scanner data sets (pannoramic viewer, version 1.15.4, 3D HISTECH Ltd., Budapest, Hungary) with pixel dimensions of 0.325 µm. Manual correction of artifacts (i.e., tissue overlaps, air bubbles, unspecific staining, dirt/fluorescent particles, and blooming) was carried out (Adobe Photoshop CS6, Adobe Systems Inc., San Jose, CA, USA) and spectral bleedthrough between different color channels was corrected using the ‘Spectral Unmixing’ plugin for imagej (version 1.51n, http://imagej.nih.gov/ij/). Image analysis was performed with mathematica (version 11.1, Wolfram Research, Inc., Champaign, IL, USA). Corrected fluorescence images were imported, split into separate color channels, and tissue masks as well as DAPI (blue channel) and proliferation marker (Ki67; green channel) masks were obtained using appropriate thresholding methods [28, 29]. The resulting masks were further cleared of very small segments to eliminate specks of fluorescent particles within nuclei. Finally, the areas of total tissue, DAPI and Ki67 masks were determined and ratios were computed. Tissue slices with apoptosis staining underwent the same procedures. Apoptosis was captured using the TRITC filter (red channel) of the digital slide scanner. Image export, manual artifact correction, spectral unmixing, image analysis, and parameter calculation were performed as described above. Numbers of analyzed images are summarized in Table S2.

### Statistical analysis of image quantification data

Statistical analysis was performed using ibm spss statistics (version 22; IBM Corp.; Armonk, New York, USA). Descriptive statistics were calculated and boxplots were generated using mathematica. Data were tested for normal distribution using the Shapiro–Wilk test and expressed as median and interquartile range. Group comparisons were performed using Kruskal–Wallis test. Significance for all tests was set at *P* < 0.05. To adjust the *P*‐values for multiple comparisons, Dunn’s *post hoc* tests were performed.

### RNA‐sequencing

Total RNA from cultivated GBM tissue slices was isolated using the miRNeasy mini Kit (Qiagen, Hilden, Germany) following the provided manufacturer’s protocol. RNA yield was measured with the Qubit 2.0 instrument (Life Technologies, Darmstadt, Germany) using the RNA Broad Range Assay. The extracted RNA was collected and stored at −80 °C until further processing. To remove genomic DNA, it was subjected to double DNAse digestion (TURBO DNA free Kit, Ambion, Thermo Fisher Scientific, Dreieich, Germany) before library preparation. RNA was quantified using a Qubit RNA‐Kit and the DeNovix instrument (Biozym, Hessisch Oldendorf, Germany). RNA quality was analyzed on a Bioanalyzer 2100 instrument (Agilent Technologies, Waldbronn, Germany). For subsequent RNA‐sequencing analyses, 200 ng of total RNA per sample was used. Library preparation was conducted using Truseq‐Stranded total RNA Sample Prep kit (Illumina, Inc, San Diego, CA, USA) according to the manufacturers' protocol. Molarity of each library was calculated and equal amounts were pooled and used for sequencing (12 pm). Sequencing was performed with 2 × 126‐bp paired‐end reads using HiSeq SBS Kit v4 chemistry on a HiSeq 2500 instrument (Illumina). 23‐26 pooled libraries (in total 98) were sequenced on 4 flow cells.

### Pre‐processing of RNA sequencing data

To facilitate the multistep analysis of the RNA sequencing datasets, we applied the workflow‐manager uap [30].

#### Primary and secondary data analysis

Demultiplexing of Illumina raw files was performed with the illumina bcl2fastq software, v.2.17.1 [31]. The paired‐end FASTQ reads were trimmed and filtered using adaptorremoval v.2.3.1 [32] with additional parameters to trim ambiguous bases (N) at 5′/3′ termini (‐‐trimns), remove low‐quality bases (‐‐trimqualities, ‐‐minquality 20) and keep reads with a minimum read length of 20bp (‐‐minlength 20). Transcript abundancy estimation of each sample was conducted using kallisto v.0.46.0 [33] by specifying a reverse stranded library. Human transcriptome FASTA file was downloaded from GENCODE (release 31 GRCh38.p12) and used to create a Kallisto index. Gene level quantifications were generated from the kallisto estimated counts per transcript using tximport v.1.18.0 [34].

#### Quality control

Sample QC was reported using fastqc v.0.11.5 [35] to assess base call accuracy, preseq v.2.0.3 [36] to evaluate the library complexity. For each sample, a subsample of 1 million trimmed paired‐end reads was randomly chosen by fastq‐sample v.0.8 [37] using default parameters. Subsamples were aligned to human reference genome GRCh38/hg38 using hisat2 v2.10 [38]. Duplication metrics were collected using picard tools v2.3.0 (http://broadinstitute.github.io/picard/) function MarkDuplicates using BAM files generated by HISAT2. Picard’s CollectRnaSeqMetrics was used to collect mapping percentages on intergenic, intronic, coding and UTR regions as well as gene body coverage. rseqc v.3.0.0 [39], was used to determine, read GC content, junction saturation, read pair inner distance, and strandness of reads. Aggregated data visualization for the secondary analysis and quality control were generated using the MultiQC [40] framework. fastq screen v.0.14 [41] in conjunction with bowtie2 [42] was conducted to assess RNA library composition (Table S3). For 13 samples, a noteworthy fraction from 30% to 75% of the subsampled reads mapped against human rRNA transcripts (Fig. S1c,d). The number of aligned reads using the Kallisto pseudoaligner ranged from 2.8 to 63.2 million (Fig. S1e). For principal component analysis (PCA), the gene counts were normalized using a variance‐stabilizing transformation as implemented in deseq2 v1.30.1 [43]. This was run with the option ‘blind = TRUE’ in order to compare samples in an unbiased manner. PCA of samples was based on the 5000 most variable genes. The variation in the first component is partly explained by samples in which an increased percentage of reads mapped against the human rRNA reference (Fig. S1f). A pairwise correlation analysis was performed between the replicates (if available) of each sample group (treated and untreated GBM tissue slices). A weaker correlation was observed in sample groups whose replicates were enriched in human rRNA (Fig. S2).

#### Sample filtering

Samples in which the subsampled reads mapped with at least 30% against the human rRNA reference or with a library size (sum of all raw counts) less than 10 million were removed from further analyses (82 out of 98 samples remained). Furthermore, only matched pairs of treated and untreated samples were analyzed. A total of 80 samples from each of 23 treated and untreated GBM tissue slices were analyzed. For each GBM tissue slice, 1–3 replicates were available.

### Differential gene expression analysis

Differential expression between treated and untreated samples was assessed with negative binomial models by using the R/Bioconductor library deseq2 v.1.30.1 [43]. An unspecific expression filter was applied to the gene count matrix. This means that at least 5 counts had to be present in at least 25% of all samples. For each gene that passed the expression filter, a generalized linear model was fitted using the formula: ~block + contrast, where block encoded the patient (to account for patient‐specific differences in gene expression) and contrast was a factor containing information on both untreated and treated for each sample. Empirical estimation of the null distribution was performed with the fdrtool r package v.1.2.16 [44] using the Wald statistic from DESeq2 as input. The estimated *P*‐values were adjusted for multiple testing with the Benjamini–Hochberg correction [45]. A gene was considered significantly differentially expressed if the FDR‐adjusted *P*‐value was < 0.05. Regularized log2‐fold‐changes (lfc) were calculated using the lfcShrink() function from the DESeq2 package to account for the variance of lfcs estimates for genes with low read counts.

For hierarchical clustering (Fig. S3) of the significantly differentially expressed genes (DEGs), variance‐stabilized gene counts were adjusted for the factor patient using the removeBatchEffect() function in the r/bioconductor limma v.3.46.0 package [46].

### Immune microenvironment analyses

Deconvolution of gene expression data was performed by TIMER [47] implemented in immunedeconv R package [48]. We filtered out those samples where the treatment strategy of the patients was not comparable to that of the GBM tissue slices. A total of 56 samples from each of 16 treated and untreated GBM tissue slices with 1–3 replicates were analyzed. Transcripts per million (TPM)‐normalized gene expression data in non‐log space were used as input for estimation of immune cell infiltrates. For all further analyses, the median of the relative abundance of immune cell types (relative infiltration scores) estimated by TIMER was calculated from GBM slice samples with more than one replicate. A Wilcoxon rank sum test for paired samples was performed to calculate the statistical significance of the estimated relative infiltration scores of immune cell types between treated and untreated GBM tissue slices. The association between standardized relative infiltration scores and the OS for untreated samples was assessed using the univariate Cox proportional hazards regression analysis. The *P*‐values and 95% Cis for Cox proportional hazard model were computed by the R function coxph() in survival v.3.2.7 R package [28].

### Pathway analysis

Gene enrichment analysis for significantly DEGs was performed using the R package clusterProfiler [29] on the WikiPathways database [49]. Significance of enrichment was assessed by a hypergeometric test and adjusted *P*‐values for multiple testing were calculated based on the Benjamini–Hochberg method (adjusted *P*‐value < 0.05). Furthermore, at least 5 significantly DEGs must be significantly enriched in the pathways.

## Results

### Tissue slices from long‐term survivors show increased apoptosis but no difference in proliferation rate after treatment

**Fig. 1:**
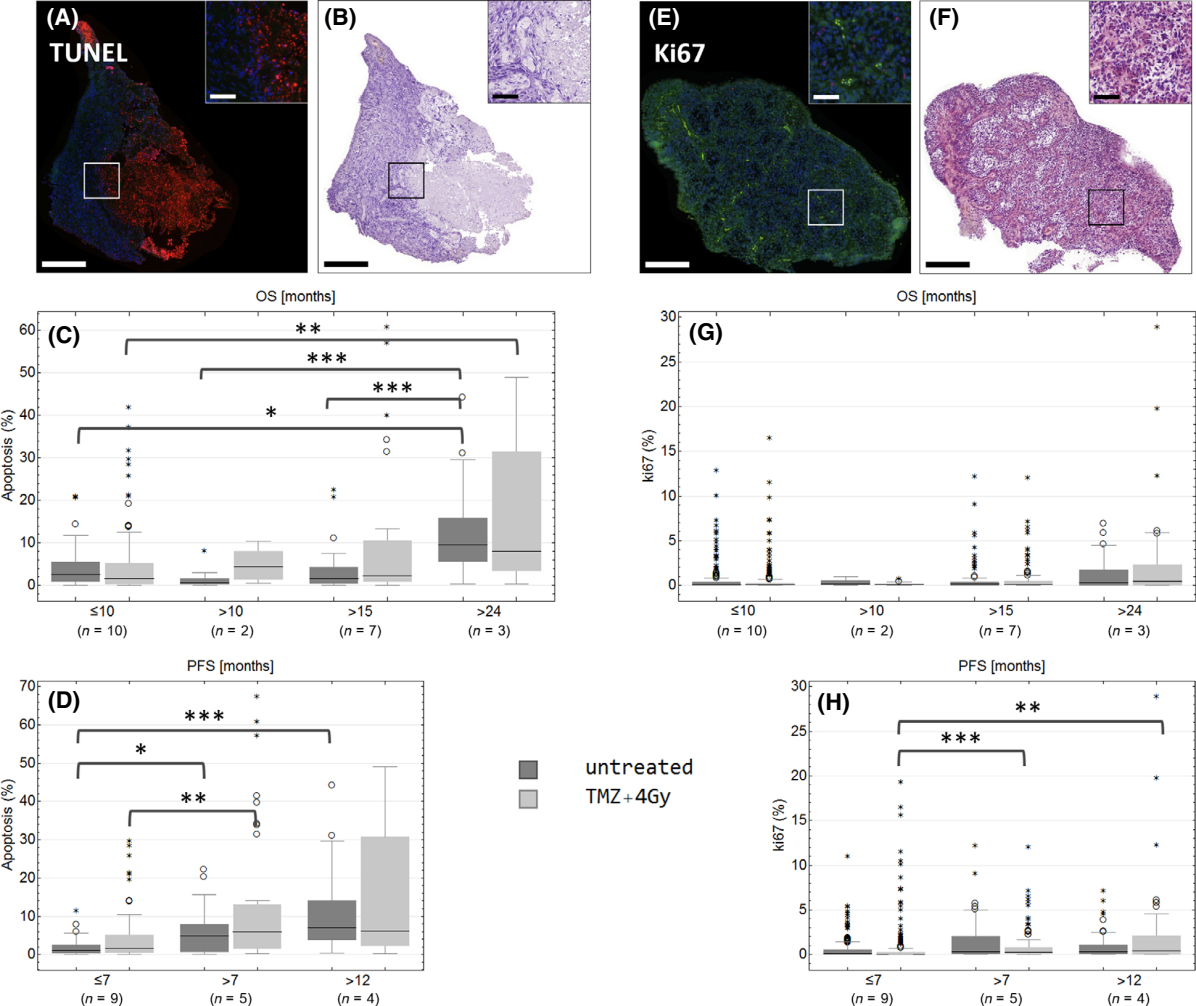
TUNEL (A–D) and Ki67 (E–H) staining in treated (light gray) or untreated (dark gray) GBM tissue slices. Treated (light gray, ‘TMZ+4 Gy’) or untreated slices (dark gray, ‘untreated’) were stained with TUNEL assay (red, A–D) or with an antibody against Ki67 (green, E‐H). Fluorescence (A, E) and bright‐field images (B, F) were recorded by a digital slidescanner. Representative images of untreated sectional samples are presented (A‐B, E‐F). For quantification, the total tissue area, DAPI‐positive nuclei area, and the Ki67‐positive or TUNEL‐positive area were determined. Samples were assorted in groups concerning OS (months) and PFS (months). Numbers of biological replicates are as follows: OS ≤ 10: *n* = 10, OS > 10: *n* = 2, OS > 15: *n* = 7, OS > 24: *n* = 3, PFS ≤ 7: *n* = 9, PFS > 7: *n* = 5, PFS > 12: *n* = 4. Outliers are marked with small circles (O) and extreme values are marked with small asterisks (*). Scale bars: 500, 100 µm in the caption. *P*‐values were adjusted by Kruskal–Wallis test with Dunn's post hoc test for multiple comparisons. Large asterisks centered above the brackets indicate significant differences: ***P ≤ 0.001, **P ≤ 0.01, *P ≤ 0.05.

To monitor treatment‐mediated effects in cultivated GBM tissue slices from human patients, immunofluorescence staining for proliferation and apoptosis was done in treated and untreated paraffin‐embedded slices (Fig. 1). Apoptosis was detected by TUNEL staining (Fig. 1A–D). Untreated slices are shown as examples (Fig. 1A,B,E,F). Fig. 1C,D Shows the quantification of apoptosis in samples sorted by the patient’s OS and progression‐free survival (PFS) in months. The following groups were defined: OS ≤ 10 months, OS > 10 months, OS > 15 months, OS > 24 months (=defined as long‐term survivors); PFS ≤ 7 months, PFS > 7 months, PFS > 12 months. Significantly increased apoptosis was found in long‐term survivors in comparison to patients with OS ≤ 10 months, both in untreated (median 9.52% vs. 2.55%, *P* = 0.014) and treated slices (8.00% vs. 1.58%, *P* = 0.008; Fig. 1C). Further, significantly higher apoptosis (9.52%) was detected in untreated samples of long‐term survivors compared to patients with OS of 10–15 months (0.62%, *P* = 0.001) and 15–24 months (1.63%, *P* = 0.001). No significant difference in apoptosis was found between treatment and control, but there was a tendency of treatment‐mediated apoptosis in slices from patients with OS longer than 10 months (Fig. 1C). Samples from patients with PFS > 7 months showed significantly higher median apoptosis in treated samples (5.96%) compared to patients with lower PFS (1.68%, *P* = 0.003; Fig. 1D). Samples from patients with PFS > 12 months showed even higher apoptotic rates in treated slices (6.16%), but without statistical significance due to the high variation within this group (*P* = 0.058; Fig. 1D). The apoptotic rate was significantly lower in untreated samples of patients with PFS ≤ 7 months (1.12%) compared to patients with PFS > 7 months (4.81%, *P* = 0.019) and patients with PFS > 12 months (6.99%, *P* = 4 × 10^−6^; Fig. 1D).

Proliferation within GBM slices was detected by antibody staining of Ki67 (Fig. 1E–H). Quantification of Ki67 in groups with different OS did not reveal any significant difference between these groups or between untreated and treated samples, but there was a slight tendency that samples from long‐term survivors had more Ki67‐positive area than the others (Fig. 1G). In general, the proliferation rate was low, treated samples from patients with PFS ≤ 7 months exhibited even lower proliferation rate (0.05%) compared to samples with PFS over 7 months (0.27%, *P* = 1.3 × 10^−5^ ) and samples with PFS > 12 months (0.39%, *P* = 0.007; Fig. 1H).

### Immune constitution in treated and untreated GBM tissue slices

**Fig. 2:**
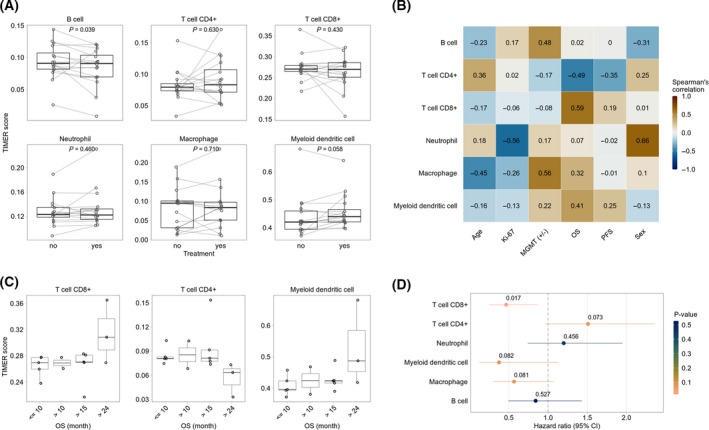
Immune microenvironment analyses. (A) Estimated relative abundance of tumor‐infiltrating immune cells using the TIMER deconvolution method in treated and untreated GBM tissue samples. Lines between dots indicate paired samples from the same patient. For patients with more than one replicate, the median relative abundance was calculated. The *P*‐values indicate the statistical significance from the Wilcoxon test for paired samples. (B) The heatmap presents Spearman’s correlation of clinical parameters and the relative abundance of tumor‐infiltrating immune cells in untreated samples. (C) Examples from correlation analysis (B) between relative abundance of immune cell types and OS. (D) Association of relative abundance of tumor‐infiltrating immune cells with overall patient survival. A univariate Cox regression was performed for untreated samples. The forest plot represents the HR and corresponding 95% confidence intervals (95%CI). The colors and the numbers above the HRs depict the statistical significance (Wald test).

For the following analysis, only the samples from patients (*n* = 15) that have been clinically treated by radiochemotherapy with TMZ were used to ensure the highest reliability of the results. Using the RNA sequencing data and analyzing it by the TIMER deconvolution method, the estimated relative abundance of tumor‐infiltrating immune cells in treated and untreated GBM tissue slices was quantified (Fig. 2A). TIMER has been developed to systematically evaluate the clinical impact of certain immune cells in cancer samples [47]. Each dot represents one tissue slice or, in case of technical/biological replicates, the median relative abundance. Treated and untreated samples of one patient are linked by lines (Fig. 2A). The relative abundance of B cells was significantly reduced in treated samples (*P* = 0.039), whereas CD8^+^ T cells, CD4^+^ T cells, neutrophils, macrophages, and dendritic cells did not exhibit any significant differences between treated and untreated slices (Fig. 2A). The relative abundance of tumor‐infiltrating immune cells was further correlated with clinical data of the patients, such as MGMT methylation state, PFS, or OS (Spearman's correlation, Fig. 2B). Samples from long‐term survivors exhibited an increased relative abundance of CD8^+^ T cells and myeloid dendritic cells, whereas the relative abundance of CD4^+^ T cells was reduced in comparison to samples from patients with lower OS (Fig. 2C). The complete results of the immune microenvironment analyses are given in Fig. S4. The hazard ratios (HR) of CD8^+^ T cells (*P* = 0.017) and macrophages (*P* = 0.081) were 0.47 and 0.57, respectively. The HR of myeloid dendritic cells (*P* = 0.082) was 0.38 (Fig. 2D). The HR of CD4^+^ T cells was 1.51 (*P* = 0.073), and those of neutrophils (*P* = 0.456) and B cells (*P* = 0.527) were 1.20 and 0.84, respectively (Fig. 2D).

### Differential gene expression between treated and untreated GBM

**Fig. 3:**
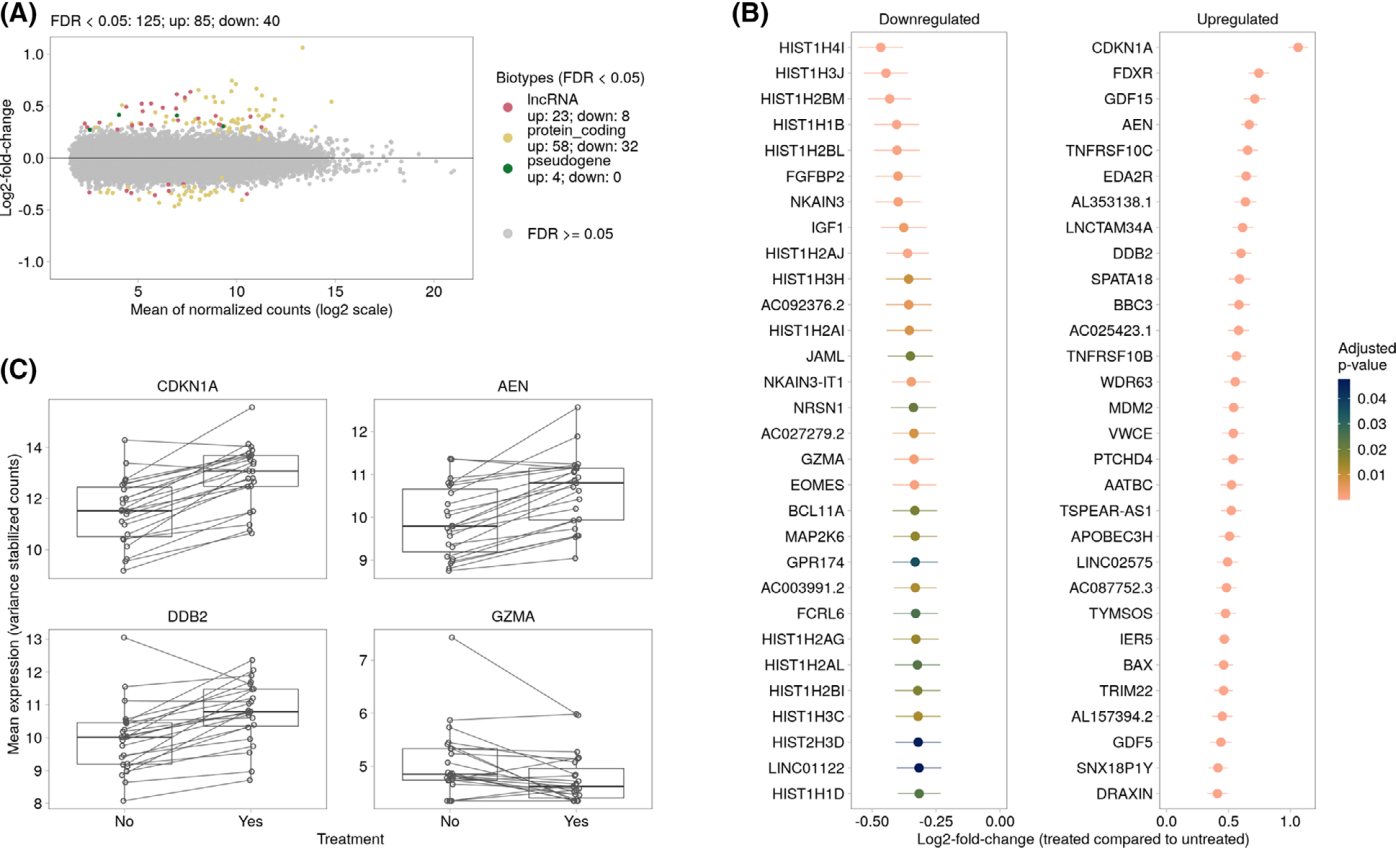
Differential gene expression analysis between treated and untreated GBM tissue samples. (A) The MA‐plot represents the relationship between normalized mean expression values and lfcs for all analyzed genes. Each dot represents a gene. Significantly DEGs (FDR < 0.05) are colored according to their gene biotype. The legend shows the number of significantly upregulated (up) and downregulated (down) genes for each gene biotype. (B) Top 20 down‐ and up‐regulated significantly regulated genes (treated compared to untreated samples). Genes are ranked by their shrunken lfcs and colored according to their adjusted *P*‐values. The vertical lines represent their estimates of standard error. (C) Examples of DEGs between treated and untreated GBM samples. Each dot represents one patient, the line links treated and untreated samples. For patients with more than one replicate, the average variance stabilized expression values were calculated.

The analysis of DEGs between treated and untreated GBM tissue slices revealed that the majority of DEGs (total: 125, up: 85, down: 40) belonged to the fraction of protein‐coding genes (up: 58, down: 32, Fig. 3A). 31 DEGs (up: 23, down: 8) could be identified as long noncoding RNA and four as pseudogenes (Fig. 3A). The top 20 up‐ and downregulated DEGs are shown in Fig. 3B in descending order of the respective lfc (Fig. 3B). A list of all DEGs is given in Table S4. Fig. 3C shows an excerpt of the significant DEGs in treated (yes, right) and untreated (no, left) GBM tissue slices for each individual patient (each dot represents one single patient). CDKN1A was the DEG with the highest lfc and was upregulated in nearly all treated samples compared to the untreated controls (median lfc = 0.89; Fig. 3C, Table S4). The same was true for DDB2 (lfc = 0.601) and AEN (lfc = 0.668), and GZMA was found to be downregulated (lfc = −0.336) in the majority of GBM samples (Fig. 3C, Table S4).

### Treatment effects on biological processes and signaling pathways

**Fig. 4:**
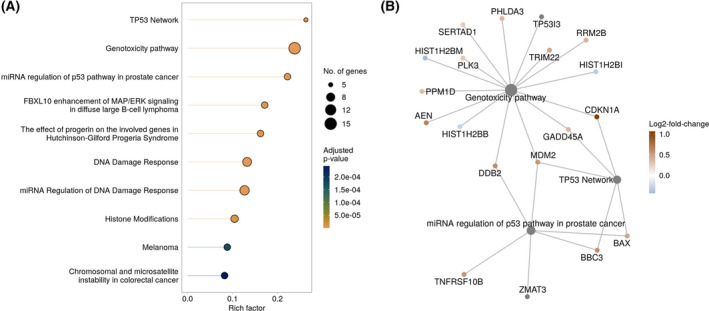
Pathway enrichment analysis of DEGs between treated and untreated GBM samples. (A) The top 10 significantly enriched pathways (FDR < 0.05) form the WikiPathways database identified by over‐representation analysis. The *x*‐axis indicates the rich factor which is the number of DEGs in the pathway divided by the number of background genes in the pathway. The size of the bubble indicates the number of involved DEGs in the pathway. The colors indicate adjusted *P*‐values of the significantly enriched pathways. (B) The linkages of genes and pathways as a network are shown. Significantly downregulated genes are shown in blue, upregulated genes in red. Shown are the identified DEGs of the three most enriched pathways.

The significant DEGs between treated and untreated GBM tissue slices were further subjected to pathway enrichment analyses (Fig. 4). The analysis of enriched signaling pathways by WikiPathways [50] demonstrated the highest enrichment of the TP53 network, the genotoxicity pathway, and the miRNA regulation of p53 pathway in prostate cancer (rich factors above 0.2, Fig. 4A). The highest number of DEGs (15) was found to be represented in the genotoxicity pathway, and 8–12 DEGs are part of DNA damage response and cancer pathways (melanoma, colorectal cancer, Fig. 4A). Figure 4B shows the top 3 most enriched pathways as a color‐coded network of the corresponding DEGs (red: upregulated, lfc > 0; blue: downregulated, lfc < 0). The network further shows the interaction of different pathways, for example, by MDM2 (mouse double minute 2 homolog) which is involved in genotoxicity pathway, TP53 network as well as miRNA regulation of p53 pathway in prostate cancer. DDB2, GADD45A, and CDKN1A play roles in genotoxicity pathway and TP53 network, and BBC3 and BAX are important players in the TP53 network as well as the miRNA regulation of p53 pathway in prostate cancer (Fig. 4b). The complete results of the WikiPathways analysis are given in Table S5.

## Discussion

Slices from patients with OS > 10 months showed increased apoptosis after treatment with radiochemotherapy (Fig. 1C), indicating a better response to the treatment and explaining longer OS. Patients with an OS ≤ 10 months exhibited lower or no apoptotic response to treatment. Slices from patients with a PFS > 12 months showed the highest apoptosis rate after treatment, indicating the highest susceptibility to cell death (Fig. 1D). Further, we did not find any significant difference in apoptosis between treated and untreated tissue slices, assuming that the detection of cell death might not be suitable for the monitoring of treatment effects. This could be due to limitations of the TUNEL assay in tumor tissue, for example, the occurrence of false‐positive signals in highly proliferative cells or the emergence of necrosis which also produces DNA single strands leading to TUNEL‐positive signals even in untreated samples [51]. The proliferation rates were significantly higher in slices from patients with a PFS > 7 and > 12 months than in the low PFS group. Proliferating tissue is more susceptible to radiation [52] and it could be shown for other tumor entities, for example, prostate cancer or oral squamous cell carcinoma, that a higher proliferation index is associated with an increased radiosensitivity of the tumor [53, 54]. This could be an explanation for the higher Ki67‐positive area in slices from patients with longer PFS, indicating a beneficial effect of the radiation. In addition, the slices from patients with an OS > 24 months exhibited higher proliferation rates. Although the difference was not statistically significant and the proliferation rates were extremely low across all samples, this observation should be handled with caution. An analysis of a larger patient cohort would be required to strengthen this finding and to validate whether Ki67 expression in tumor slices could serve as a predictive marker for radiosensitivity in GBM.

For the implementation of the TIMER deconvolution method, only samples from patients clinically treated with radiation therapy and TMZ have been used to increase the reliability of the correlation with patient survival data. The analysis of tumor‐infiltrating immune cells revealed a slight reduction of B cells after treatment. The populations of CD4^+^ and CD8^+^ T cells, neutrophils, macrophages, and dendritic cells were not affected by treatment indicating no effect of the relative abundancies of these cell types on the OS of patients (Fig. 2A). B cells have been shown to play a crucial role in the development of an inflammatory environment which promotes carcinogenesis [55]. Therefore, a reduction of B cells after treatment could provide a hint of reduced tumor‐promoting inflammation within these samples. It was further shown that the relative abundance of CD8^+^ T cells was increased in samples from patients with OS > 24 months (Fig. 2C). Cytotoxic T lymphocytes have been demonstrated to form immunological synapses with tumorigenic cells in GBM thereby suggesting a role in antitumor immune responses and tumor clearance [56]. This could also explain the higher apoptosis in slices from long‐term survivors, in both treated and untreated tissue (Fig. 1C). Further, it was shown for various cancers that a local infiltration of CD8^+^ T cells into the tumor area was correlated with a favorable prognosis [57, 58]. Dendritic cells are well‐established antigen‐presenting cells and are crucial for the activation of T lymphocytes [59]. In the context of cancer, dendritic cells prime cytotoxic T cells via antigen presentation on MHC‐I molecules and thereby enable them to specifically target tumor cells [60, 61]. A higher proportion of dendritic cells within samples from patients with OS > 24 months is in line with the higher proportion of CD8^+^ T cells (Fig. 2C).

The DEG with the highest median lfc in treated GBM slices was CDKN1A (p21). p21 is known to regulate the cell cycle and inhibit tumor growth. In accordance with that, expression of p21 leads to induction of apoptosis in GBM cells [62]. In GBM‐derived cell lines, it was found that a lack of p53 resulted in the failure of apoptosis induction, suggesting a key mechanism to radioresistance which is often observed in GBM [62]. p53 mutations are very common among GBM and approximately 85% of all GBMs exhibit a deregulation in p53 signaling [11]. In the study presented here, an enrichment of genes associated with miRNA regulation of p53 pathway in prostate cancer and the TP53 network in general was observed (Fig. 4) which gives a hint that p53 signaling has been affected by radiochemotherapy. We further found a significant upregulation of TP53I3 (tumor protein p53 inducible protein 3; lfc = 0.352, Table S4), the gene encoding for tumor protein p53 inducible protein 3 (PIG3). PIG3 expression is suppressed in GBM tissue compared to normal tissue and a higher expression is associated with a better prognosis as well as longer OS in GBM patients [63]. An increased expression in GBM slices after treatment indicates a response to the therapy.

GZMA, the gene encoding granzyme A, is mainly expressed upon activation of cytotoxic T cells and leads to apoptosis through activation of caspases [64]. CD8^+^ T cells and natural killer (NK) cells have the ability to kill cancer cells by overexpressing GZMA and perforin 1 [65]. Further, it was found for various cancer types that the presence of effector T cells within tumors is strongly associated with a better prognosis of the patient [66, 67, 68]. In GBM, a study revealed a better outcome for patients with lower expression levels of GZMA [69]. In our dataset of GBM patient tissues, the expression of GZMA was decreased upon treating the tissue slices with radiochemotherapy, indicating a beneficial effect of the therapy (Fig. 3C). However, an alteration in the NK or CD8^+^ T cell population after treatment could not be detected (Fig. 2). This could be due to the restricted time point of the analysis when mRNA expression changes are already detectable, but an adjustment of cell populations would take some more time after the initiation of treatment.

AEN (apoptosis‐enhancing nuclease) is induced by p53 and is regulated by its phosphorylation status upon DNA damage [70], for example, caused by irradiation. AEN, as a proapoptotic p53‐dependent target gene, was further shown to be induced by irradiation in U251 MG GBM cells [71]. The upregulation of AEN in radiochemotherapy‐treated GBM slices therefore is in line with the upregulation of CDKN1A as well as the treatment‐mediated enrichment of the p53 network.

DDB2 (DNA damage binding protein 2) is one of the key DNA repair proteins which is assumed to have tumor‐suppressing features and contribute to better treatment responses in tumors [72]. In GBM, an association of higher DDB2 expression with a better prognosis could be demonstrated. Concomitantly, patients with worse prognosis exhibited lower DDB2 expression [73]. In the GBM tissue slices, an increase of DDB2 expression was detected after treatment, being in line with the studies mentioned before.

One of the major problems of working with GBM tissue freshly resected from patients is the highly varying tissue quality, the limited amount of tissue and thus the lack or low number of biological replicates per experiment. Furthermore, high intra‐ as well as inter‐tumoral heterogeneity can be observed. Another drawback is the missing opportunity to compare brain tumor tissue with healthy tissue from the same patient. Despite these limiting factors, we could show that the culture model combined with RNA sequencing is a suitable model to monitor treatment‐mediated effects in GBM tissue slices on a transcriptional level. Interestingly, it was more difficult to reproduce these effects on a histological level by immunofluorescent staining. This could be due to the restricted time frame of the experimental setting where transcriptional changes can be seen early after onset of treatment, while changes at the protein or cellular level would take longer to manifest.

In conclusion, the study presented here, reproduces former studies, showing that GBM tissue slice cultures are suitable for RNA sequencing and automated histology, at a larger scale. The model system is now further improved by the correlation of the collected experimental data with the clinical course of each individual patient. At this point, it should be mentioned that, in order to merge experimental with clinical data regarding OS and PFS, the cultures had been treated and analyzed several months (years) before clinical data were available. The rational for this approach was to identify molecular and physiological characteristics of the primary tumor and its response to therapy that could be used as valuable markers to predict outcome. To investigate potential therapeutic targets, predictive biomarkers, reasons for resistance to therapy, or genetic predispositions to develop GBM, a larger patient cohort should be analyzed as the general expression patterns in these samples seem to be unique for every single patient.

## Conflict of interest

The authors declare no conflict of interest.

## Author contributions

IB, FG, FR, FH, and JM managed the funding and conceptualization of the project and provided resources. SH, AH, NK, and DL conducted the experiments and collected data. SH, MR, KW, KR, and CB analyzed study data. CR and JM performed surgery. WM performed neuropathological diagnosis. SH, MR, and KW wrote the initial manuscript. All authors reviewed the manuscript.

## Supporting information


**Fig. S1.** Quality Control of samples with respect to sequencing library composition, read alignment and sample variance. Following adapter trimming, each FASTQ file was assessed for average per base (a) and per sequence (b) quality as measured by Phred score. Samples with sequencing depth < 50000 were removed and are not shown in (c–f). (c) To assess the sequencing library composition, each sample was subsampled to randomly 1 million trimmed paired‐end reads. FastQ Screen in conjunction with bowtie2 was conducted to detect possible contamination like for example bacteria and overrepresented fractions of RNA species like human rRNA. The y‐axis depicts the percentage of first reads for each sample that aligned against the references from Table S3. Reads are classified into four distinct types indicating reads uniquely mapping in one sequence reference (one hit in in one reference), reads with multiple mappings in one sequence reference (multiple hits in one reference), reads uniquely mapping in distinct sequence references (one hit in multiple references) and reads with multiple mappings in distinct sequence databases (multiple hits in multiple references). (d) Subsampled reads were mapped iteratively against the RNAmmer database v1.2, human rRNA and the human genome assembly GRCh38/hg38. Human rRNA reads are divided into sense, which resembles endogenous rRNA and antisense RNA which indicate rRNA antisense probes from the rRNA depletion step. Samples mapped with at least 30% against the human rRNA reference depicted with asterisks. (e) Quality assessment of read alignment. Number of fragments aligned or not aligned to the human hg38 reference transcriptome using the Kallisto pseudoaligner. Samples mapped with at least 30% against the human rRNA reference depicted with asterisks. (f) Principal component analysis (PCA) of variance‐stabilized counts based on the 5000 most variable genes. The upper plot depicts first and second principal components, the bottom plot the second and third principal component. The upper PCA is colored by library size (sum of all raw count for each sample) and shaped by treatment. The bottom PCA is colored by binned percentages of reads mapped against the human rRNA reference.

**Fig. S2.** Correlation analysis between replicates of each sample. For each sample group (treat and untreated GBM tissue slices), normalized expression levels were correlated between replicates (if available). The color of each hexagonal bin in the scatter plot represents the number of genes overlapping at that position. The Coefficient of Determination (R2) and estimated regression model are shown. Axis labels are colored by binned percentages of reads mapped against the human rRNA reference. The last integer in the axis labels indicate the replicate. Treated samples are labeled by ‘T’.

**Fig. S3.** Hierarchical clustering of treated and untreated GBM tissue samples based on significantly DEGs. Euclidean distance clustering and complete linkage was applied to visualize similarity between samples. Each column represents a sample, and each row represents a gene. Variance stabilized expression values for each gene were *z*‐score standardized.

**Fig. S4.** Relationship between relative abundance of immune cell types estimated using the TIMER deconvolution method and clinical parameters. Relationship between relative abundance of immune cell types estimated using the TIMER deconvolution method and clinical parameters.

**Table S1.** Summarized data of the patient cohort.

**Table S2.** Numbers of analyzed images per condition and experiment.

**Table S3.** References used for FastQ Screen.

**Table S4.** Complete list of significantly DEGs (FDR < 0.05) between treated and untreated samples.

**Table S5.** Complete over‐representation analysis (ORA) by WikiPathways.

## Data Availability

The deep sequencing datasets generated and analyzed during the current study are available in the GEO repository GSE179649. The histological datasets generated during the study are available from the corresponding author on reasonable request.
