# Supplementary figures and images for: RNA sequencing of glioblastoma tissue slice cultures reveals the effects of treatment at the transcriptional level
Source: FEBS Open Bio. 2021 Dec 29;12(2):480–93. doi: 10.1002/2211-5463.13353 (PMC8804611; doi:10.1002/2211-5463.13353)

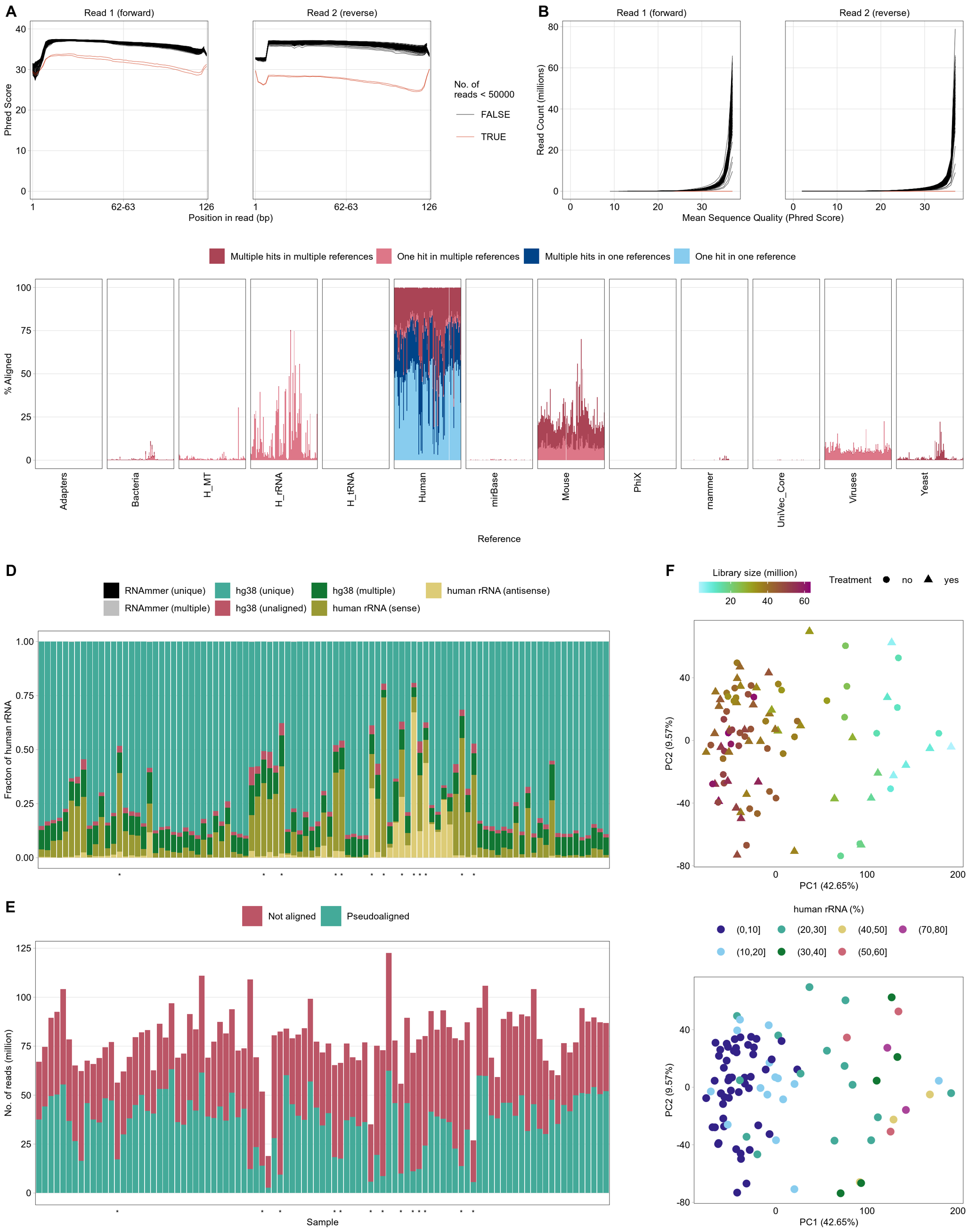

Supplement: Supplementary file 1 — Fig. S1. Quality Control of samples with respect to sequencing library composition, read alignment and sample variance. Following adapter trimming, each FASTQ file was assessed for average per base (a) and per sequence (b) quality as measured by Phred score. Samples with sequencing depth < 50000 were removed and are not shown in (c–f). (c) To assess the sequencing library composition, each sample was subsampled to randomly 1 million trimmed paired‐end reads. FastQ Screen in conjunction with bowtie2 was conducted to detect possible contamination like for example bacteria and overrepresented fractions of RNA species like human rRNA. The y‐axis depicts the percentage of first reads for each sample that aligned against the references from Table S3. Reads are classified into four distinct types indicating reads uniquely mapping in one sequence reference (one hit in in one reference), reads with multiple mappings in one sequence reference (multiple hits in one reference), reads uniquely mapping in distinct sequence references (one hit in multiple references) and reads with multiple mappings in distinct sequence databases (multiple hits in multiple references). (d) Subsampled reads were mapped iteratively against the RNAmmer database v1.2, human rRNA and the human genome assembly GRCh38/hg38. Human rRNA reads are divided into sense, which resembles endogenous rRNA and antisense RNA which indicate rRNA antisense probes from the rRNA depletion step. Samples mapped with at least 30% against the human rRNA reference depicted with asterisks. (e) Quality assessment of read alignment. Number of fragments aligned or not aligned to the human hg38 reference transcriptome using the Kallisto pseudoaligner. Samples mapped with at least 30% against the human rRNA reference depicted with asterisks. (f) Principal component analysis (PCA) of variance‐stabilized counts based on the 5000 most variable genes. The upper plot depicts first and second principal components, the bottom plot the se [file FEB4-12-480-s008.tif]

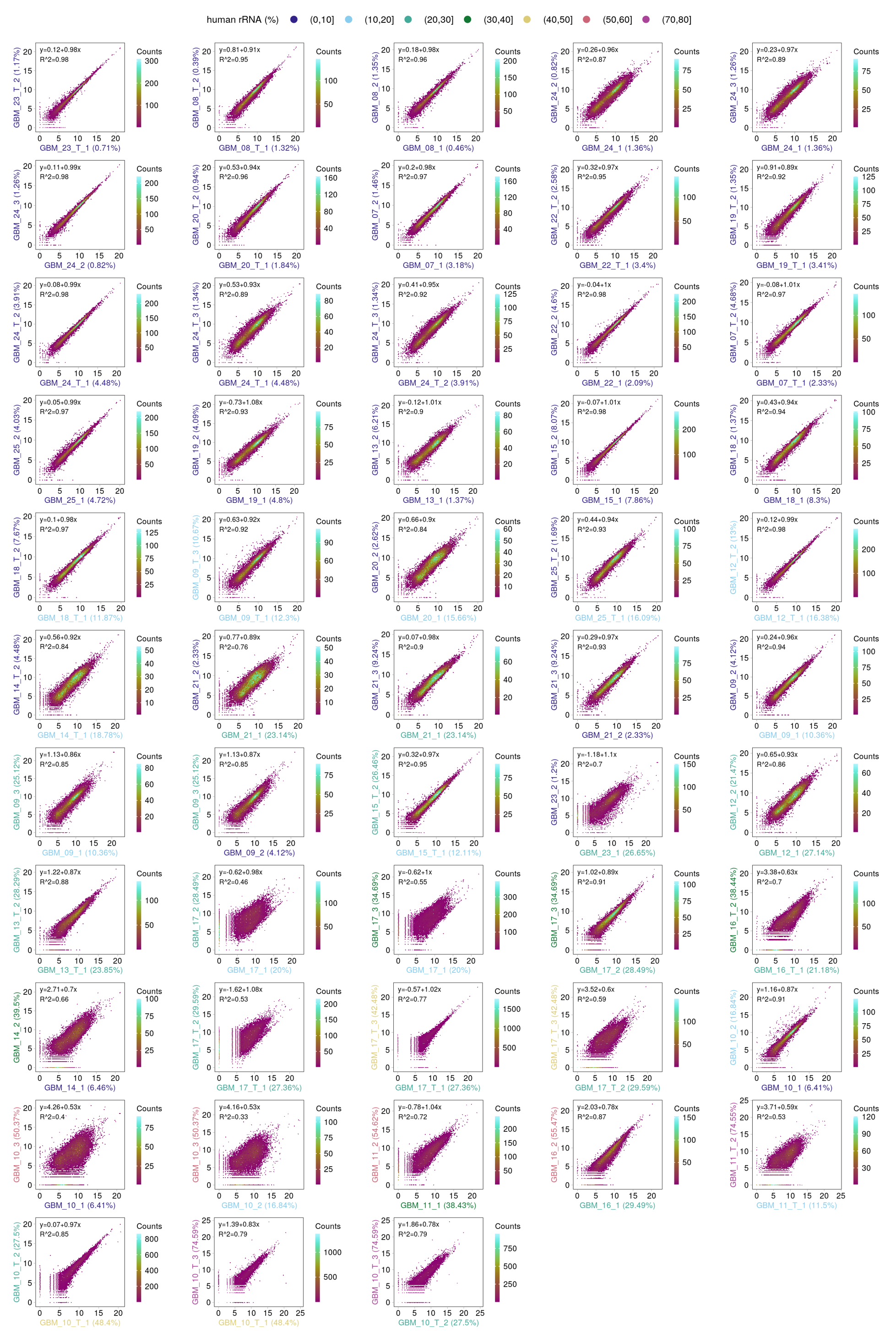

Supplement: Supplementary file 2 — Fig. S2. Correlation analysis between replicates of each sample. For each sample group (treat and untreated GBM tissue slices), normalized expression levels were correlated between replicates (if available). The color of each hexagonal bin in the scatter plot represents the number of genes overlapping at that position. The Coefficient of Determination (R2) and estimated regression model are shown. Axis labels are colored by binned percentages of reads mapped against the human rRNA reference. The last integer in the axis labels indicate the replicate. Treated samples are labeled by ‘T’. [file FEB4-12-480-s007.tif]

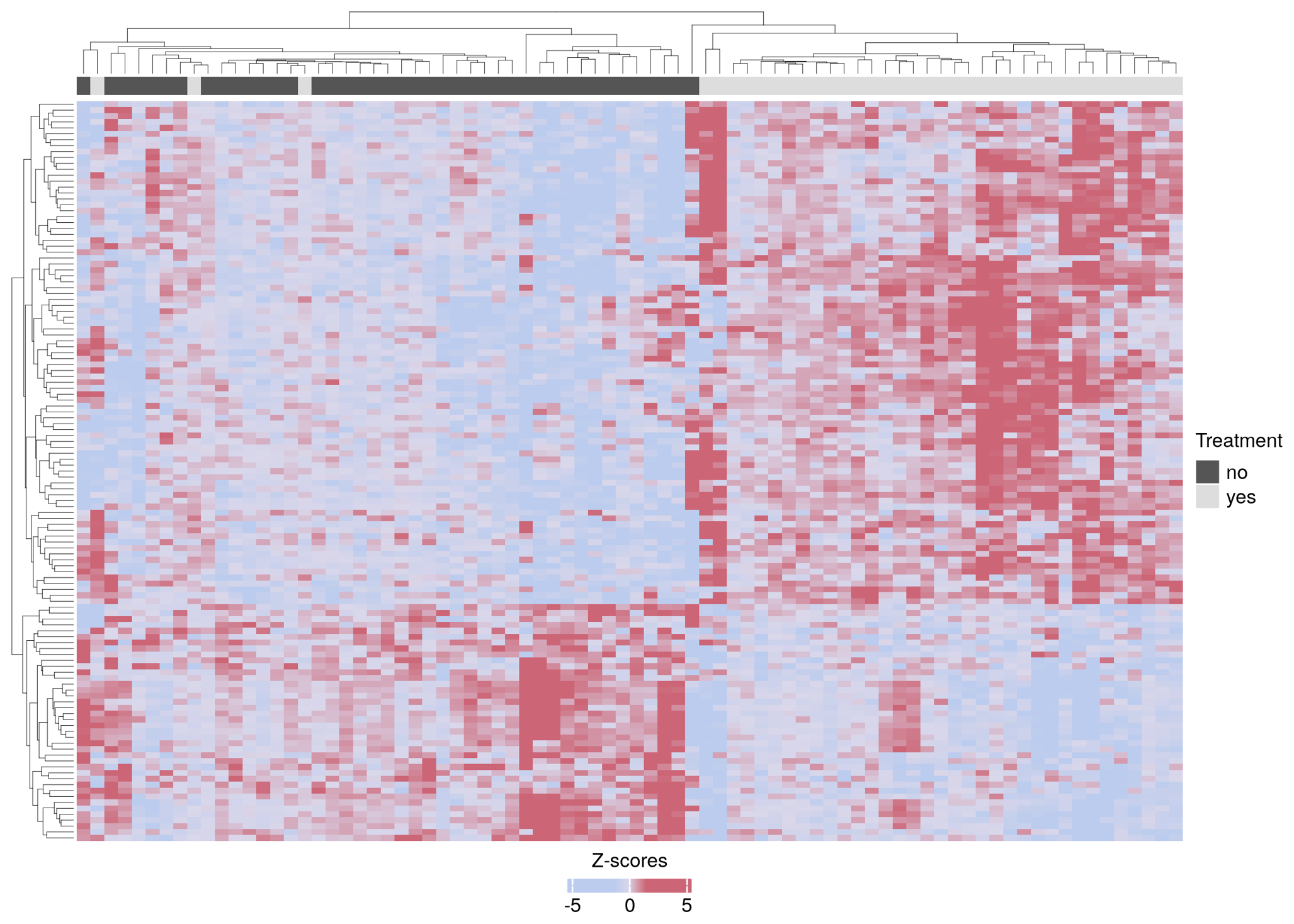

Supplement: Supplementary file 3 — Fig. S3. Hierarchical clustering of treated and untreated GBM tissue samples based on significantly DEGs. Euclidean distance clustering and complete linkage was applied to visualize similarity between samples. Each column represents a sample, and each row represents a gene. Variance stabilized expression values for each gene were z‐score standardized. [file FEB4-12-480-s009.tif]

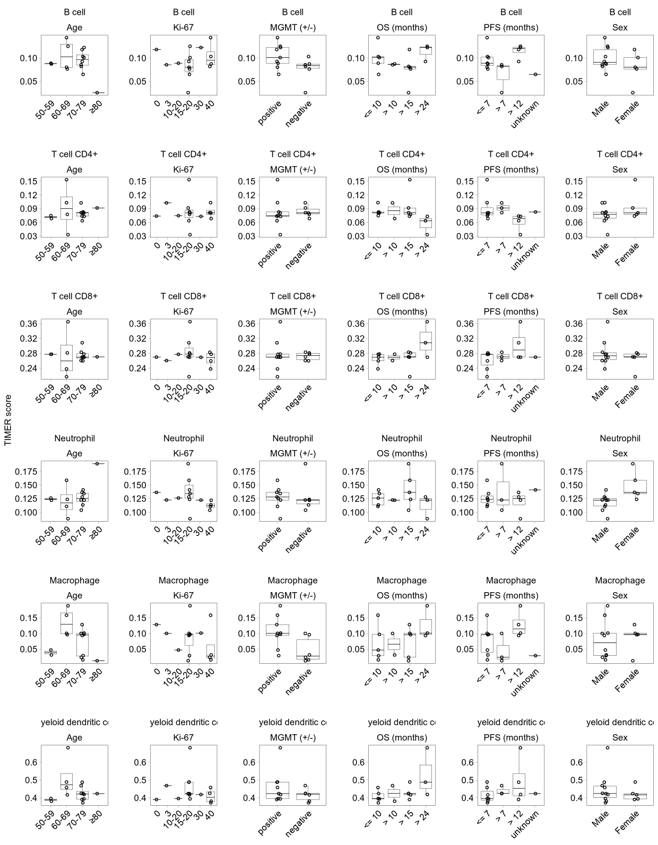

Supplement: Supplementary file 4 — Fig. S4. Relationship between relative abundance of immune cell types estimated using the TIMER deconvolution method and clinical parameters. Relationship between relative abundance of immune cell types estimated using the TIMER deconvolution method and clinical parameters. [file FEB4-12-480-s001.tif]
